# HIV-1 Autologous Antibody Neutralization Associates with Mother to Child Transmission

**DOI:** 10.1371/journal.pone.0069274

**Published:** 2013-07-17

**Authors:** Elly Baan, Anthony de Ronde, Martijn Stax, Rogier W. Sanders, Stanley Luchters, Joseph Vyankandondera, Joep M. Lange, Georgios Pollakis, William A Paxton

**Affiliations:** 1 Laboratory of Experimental Virology, Department of Medical Microbiology, Centre for Infection and Immunity Amsterdam (CINIMA), Academic Medical Centre of the University of Amsterdam, Amsterdam, The Netherlands; 2 Department of Microbiology and Immunology, Weill Medical College of Cornell University, New York, New York, United States of America; 3 International Antiviral Therapy Evaluation Center (IATEC), Amsterdam, The Netherlands; 4 Centre Hospitalier Universitaire de Kigali (CHUK) and Belgian Technical Cooperation, Kigali, Rwanda; University of California San Francisco, United States of America

## Abstract

The HIV-1 characteristics associated with mother to child transmission (MTCT) are still poorly understood and if known would indicate where intervention strategies should be targeted. In contrast to horizontally infected individuals, exposed infants possess inherited antibodies (Abs) from their mother with the potential to protect against infection. We investigated the HIV-1 gp160 envelope proteins from seven transmitting mothers (TM) whose children were infected either during gestation or soon after delivery and from four non-transmitting mothers (NTM) with similar viral loads and CD4 counts. Using pseudo-typed viruses we tested gp160 envelope glycoproteins for TZM-bl infectivity, CD4 and CCR5 interactions, DC-SIGN capture and transfer and neutralization with an array of common neutralizing Abs (NAbs) (2F5, 2G12, 4E10 and b12) as well as mother and infant plasma. We found no viral correlates associated with HIV-1 MTCT nor did we find differences in neutralization with the panel of NAbs. We did, however, find that TM possessed significantly higher plasma neutralization capacities than NTM (*P* = 0.002). Furthermore, we found that *in utero* (IU) TM had a higher neutralization capacity than mothers transmitting either *peri* **-** *partum* (PP) or via breastfeeding (BF) (*P* = 0.002). Plasma from children infected IU neutralized viruses carrying autologous gp160 viral envelopes as well as those from their corresponding mothers whilst plasma from children infected PP and/or BF demonstrated poor neutralizing capacity. Our results demonstrate heightened autologous NAb responses against gp120/gp41 can associate with a greater risk of HIV-1 MTCT and more specifically in those infants infected IU. Although the number of HIV-1 transmitting pairs is low our results indicate that autologous NAb responses in mothers and infants do not protect against MTCT and may in fact be detrimental when considering IU HIV-1 transmissions.

## Introduction

According to the 2011 UNAIDS Progress report an estimated 2.7 million people worldwide were newly infected with HIV-1 in 2010. Of this 390,000 were infants with the majority resulting from mother-to-child transmission (MTCT). Known maternal risk factors associated with MTCT are high plasma viral loads, low CD4 T-cell numbers coinciding with advanced maternal immune deficiency and prolonged labor [1]. In populations where replacement feeding is not feasible it has been estimated that 41% of MTCT occur *in utero* (IU), 20% *peri-partum* (PP) and the remaining 39% during prolonged breastfeeding (BF) [2]. The majority of transmissions are found in regions where antiretroviral therapy availability is limited, such as sub-Saharan Africa (UNAIDS Progress report 2011) and specifically regions where HIV-1 subtype A and C predominate, including the growing number of infections in Russia [3]. Little is known regarding mechanisms determining risk of MTCT but better understanding of such events will be critical in designing effective means to limit transmissions.

As seen with HIV-1 sexual transmission the established viruses in MTCT predominantly utilize the CCR5 coreceptor (R5) for cell entry and rarely CXCR4 (X4) [4], [5]. Earlier studies have indicated that HIV-1 transmissions are initiated by a single or limited number of donor viruses, often a minor variant, indicating a bottleneck in transmission or selective outgrowth of transmitted variants [6], [7]. Much attention has focused on defining the genetic and phenotypic properties of the HIV-1 gp120/gp41 envelope glycoprotein (Env) of HIV-1 since this directs the receptor and coreceptor interactions that determine infection. The Env is also the major target of the host immune response and induces binding antibodies (Abs), some of which are neutralizing (NAbs) that can control or prevent infection [8]–[10]. There has been much speculation that viral fitness may determine MTCT with some studies showing that viruses from transmitting mothers (TM) possess higher replication capacities than viruses generated from non-transmitting mothers (NTM) [11], [12]. Two studies found no difference in infectivity between mothers and children’s *env* clones tested in a single-cycle assay [13], [14]. A study comparing Env pseudo-typed viruses generated from subtype C infected MTCT pairs demonstrated that Env from children have a higher replication capacity than Env from the mothers which is V1V5 restricted [15]. Additionally, no differences were found between transmitted and non-transmitted viruses for their capacity to utilize CD4 or the CCR5 corceptor [13], [14], [16].

Studies of adult HIV-1 transmission pairs in Africa have shown that viruses undergoing horizontal transmission possess Env genotypes with shorter variable loops and fewer numbers of potential N-linked glycosylation sites (PNGS) which can associate with the development of anti-HIV-1 Ab responses [17]. Correlations between variable loop length and number of putative PNGS have been reported for MTCT. In some studies fewer Env PNGS are found in the transmitted viral variants whilst other studies do not find differences in total number but have found the position of the PNGS to associate with risk of transmission [14], [18]–[20].

In MTCT Abs are present in the exposed child having been passed from the mother. The common perception is that these Abs protect against HIV-1 infection or select variants undergoing transmission. In agreement with this notion, animal models indicate that Abs can reduce or prevent MTCT [21]–[23]. Reports on human mother child pairs have shown better neutralization by NTM than by TM suggesting a protective role by Abs [24], [25]. Others report better neutralization by TM or find no differences between TM and NTM [12], [14], [18], [24], [26]–[29]. Neutralization resistance in children against mother’s plasma or serum has been reported suggesting transmission of neutralization escape mutants, but, in contrast, sensitivity for neutralization by plasma of the mother has also been found [18], [24], [26], [27], [30], [31]. These discrepancies may depend on differences in viral subtype, mode of transmission, timing of transmission, timing of sampling or the selective study of autologous versus non-autologous viruses. Although the role of maternal NAbs in MTCT is controversial trials with HIV-Ig have been conducted. One demonstrated protection against IU transmission whilst the other revealed a significant increase in the number of infections at birth and 2 weeks after delivery in the treated versus untreated group [32], [33].

IgG transport from the placenta to the fetus during gestation is mediated by the FcRn receptors expressed on the syncytiotrophoblast followed by transcytosis [34]. The FcRn receptor is also expressed on intestinal mucosa and functions in the uptake of IgG from breast milk. Although the FcRn receptor binds monomeric IgG more avidly than aggregated or immunocomplexed IgG the mechanism may provide a route whereby HIV-1/anti-HIV-1 IgG complexes can be transferred to the fetus [35]. Total IgG binding to gp160, gp120 glycoproteins and/or peptides derived from V3 or gp41 was measured in TM and NTM individuals with better binding found in the TM, which may be in line with heightened enhancement to transcytosis of HIV-1 via HIV/IgG complexes [12],[36]–[38]. In addition, it has been demonstrated in studies utilizing a trophoblast monolayer model mimicking the barrier between the placenta and fetal blood that the fusion of HIV-1 infected PBMCs, monocytes and macrophages at the apical side of the monolayer can be followed by HIV-1 transcytosis to the lateral side [35], [39].

HIV-1 MTCT requires passage of virus across a mucosal barrier, be it via the amniotic fluid during IU, from blood and vaginal secretions during IP and/or virus present in breast milk during feeding. Experiments using *ex vivo* fetal oral and intestinal tissue model systems have indicated that infectious cell free HIV-1 can traverse the oral as well as intestinal mucosa and that HIV-1 infected macrophages can also transmigrate across these surfaces [40]. To a lesser extent HIV-1 infected lymphocytes were able to cross the intestinal epithelium and establish infection. It has been widely postulated that HIV-1 mucosal infection can be heightened through the interaction of virus with an array of C-type lectins, including DC-SIGN, expressed on dendritic cells (DCs) lying below the mucosal epithelium [41]–[43]. One study associating a specific DC-SIGN genetic polymorphism with risk of HIV-1 infection indeed suggests that this molecule has a role to play in viral transmission [44]. DC-SIGN allows for the capture of virus and its heightened transfer to CD4^+^ lymphocytes, termed *trans*-infection. We have shown that this mechanism can be enhanced when virus is coated with Abs and taken up via the Fc receptor on DCs and that the neutralization effects of numerous Abs can be negated through such an interaction [45]. A number of glycoproteins present in human bodily secretions, including milk, have been reported to bind DC-SIGN and block HIV-1 capture [46]–[49]. One such molecule, bile-salt stimulated lipase (BSSL) from milk, is highly polymorphic with the variant forms differentially inhibiting viral capture and infection of CD4^+^ lymphocytes [49]. How well virus interacts with DC-SIGN or escapes natural inhibitors may provide the virus with a phenotypic advantage in MTCT.

Here we study Env pseudo-typed viruses generated from TM, NTM and their children to identify phenotypes that associate with the risk of MTCT. From this analysis we conclude that strong autologous neutralization activity can associate with risk of transmission indicating that high gp120/gp41 NAb responses may in fact be detrimental.

## Results

### Selection of *env* Clones for Study

**Figure 1:**
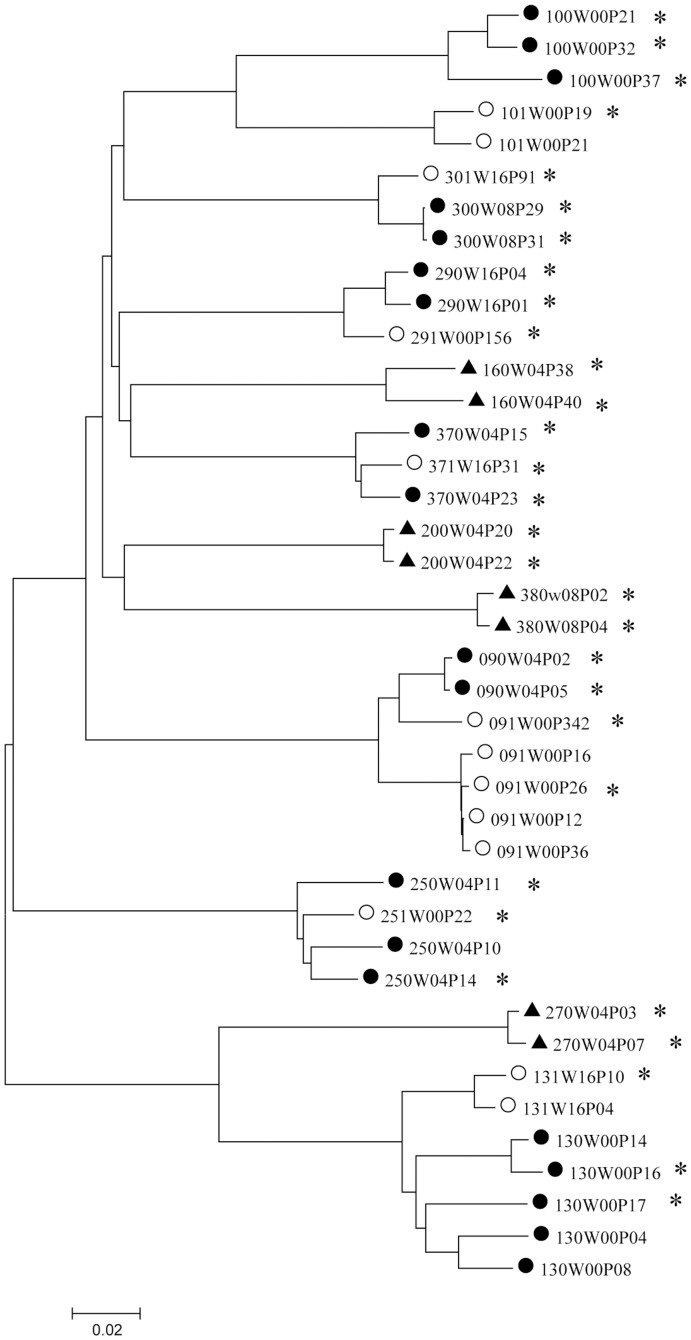
Phylogenetic analysis of the gp160 envelope region. The analysis was performed using the Neighbor-Joining (N-J) method of MEGA version 4. Positions containing an alignment gap were included for pair-wise sequence analysis. TM depicted by closed circles, IC by open circles and NTM by triangles. The clones selected for further analyses are indicated with an asterix.

From a cohort of 30 pregnant HIV-1 positive women from Rwanda we generated Env pseudo-typed viruses from seven MTCT pairs (090/091, 100/101, 130/131, 250/251, 290/291, 300/301 and 370/371), four infected IU and three PP or BF (Table 1). We included a group of four NTM (160, 200, 270 and 380) infected with similar viral loads and CD4 counts. We generated pseudo-typed viruses expressing whole gp160 Env derived from plasma of mothers and children and screened for infectivity on TZM-bl cells. The *env* clones that gave rise to infectious pseudo-typed viruses were sequenced and subjected to phylogenetic analysis (Fig. 1). This analysis confirmed the relatedness of the virus circulating in the respective mother child pairs. One transmission pair (130/131) was infected with subtype C virus, five pairs were infected with subtype A (090/091, 100/101, 290/291, 300/301 and 370/371) and one pair (250/251) carried an AC recombinant. Three NTM (160, 200, and 380) harbored a subtype A virus and one (270) subtype C. Earlier analysis on this cohort showed that six out of the seven pairs demonstrated transmission of a single virus variant, whilst pair 90/91 showed transmission of two variants [20] (Fig. 1). Since the viral population in each child was homogenous we selected one clone from each for analysis, with exception of child 091 where both variants were analyzed. For each TM and NTM we randomly selected two variants. From three TM and two NTM the clones were shown to closely cluster and from mothers 300, 290, 370 and 090 only two variants were available and both were utilized. From mother 100 we selected a third variant (100P37) based on sequence prediction indicating it to be a R5/X4 dual-tropic virus. We investigated the constructed Env pseudo-typed viruses from NTM, TM and IC for properties such as entry, receptor and coreceptor usage, interaction with DC-SIGN as well as neutralization sensitivity.

**Table 1:** Characteristics of transmitting mothers, non-transmitting mothers and children.

Subject	TM/NTM	Subtype	Viral load (screen) RNA copies/ml	Viral load (wk16) RNA/copies/ml	CD4 count (wk0) cells/µl	Time-point clones (week)	Time-point late plasma (week)	Subject	PCR Wk0	Viral load (wk16) RNAcopies/ml	Time-point clones	Time-point plasma
90	TM	A	131,000	97,859	250	4	17	**91**	Pos	750,000	0	16
100	TM	A	208,000	sna	275	0	16	**101**	Pos	sna	0	sna
130	TM	C	61,900	53,031	595	0	18	**131**	Neg	7,590	16	16
250	TM	A/C	8,820	15,945	206	4	18	**251**	Pos	269,000	0	16
290	TM	A	1,570	124,084	1168	16	18	**291**	Pos	531,000	0	16
300	TM	A	7,130	174,000	770	8	18	**301**	Neg	747,000	16	16
370	TM	A	5,430	28,471	289	4	16	**371**	Neg	144,000	16	16
160	NTM	A	84,400	sna	164	4	16	**161**	–	–	–	sna
200	NTM	A	7,100	5,848	370	4	18	**201**	–	–	–	16
270	NTM	C	19,700	251,994	387	4	16	**271**	–	–	–	16
380	NTM	A	7,840	56,253	602	8	18	**381**	–	–	–	16

TM: Transmitting mothers, NTM: non-transmitting mother, Pos: positive, Neg: negative, sna: sample not available.

### Env Pseudo-typed viruses from TM and NTM more Infectious than viruses from Children

**Figure 2:**
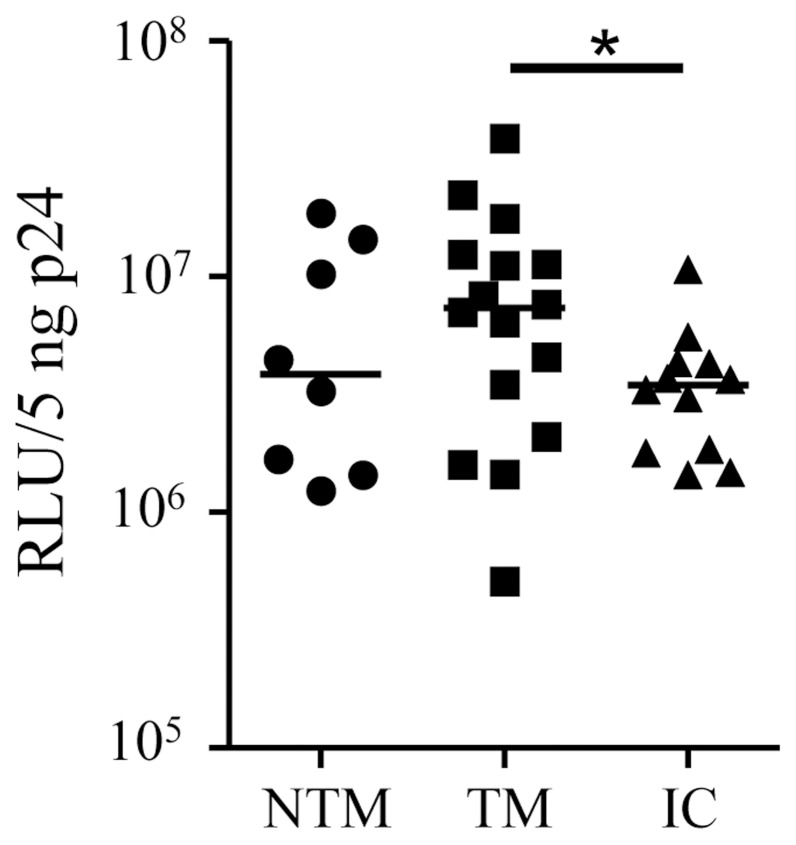
Infectivity of TZM-bl cells with Env pseudo-typed viruses. Viruses were tested from NTM, TM and infected children (IC) with results expressed in relative luciferase units (RLU). *Probability that the groups are similar is <0.05.

We tested the infectivity of all pseudo-typed viruses from NTM, TM and children in the TZM-bl single-cycle infection assay. Limiting dilutions of the viral stock was performed to determine that the readings were within the linear range when testing up to 5 ng CA-p24 input. The infectivity median was determined by measuring the Relative Luciferase Units (RLU) normalized to the amount of capsid p24 (CA-p24) (Fig. 2). We found no difference between viruses of NTM and TM but the children’s virus clones showed significantly lower entry efficiency than the TM (*P = *0.04) indicating that high infectivity is not associated with virus selection during transmission.

### No Differences in virus Affinity for CD4 between TM, NTM or Children

**Figure 3:**
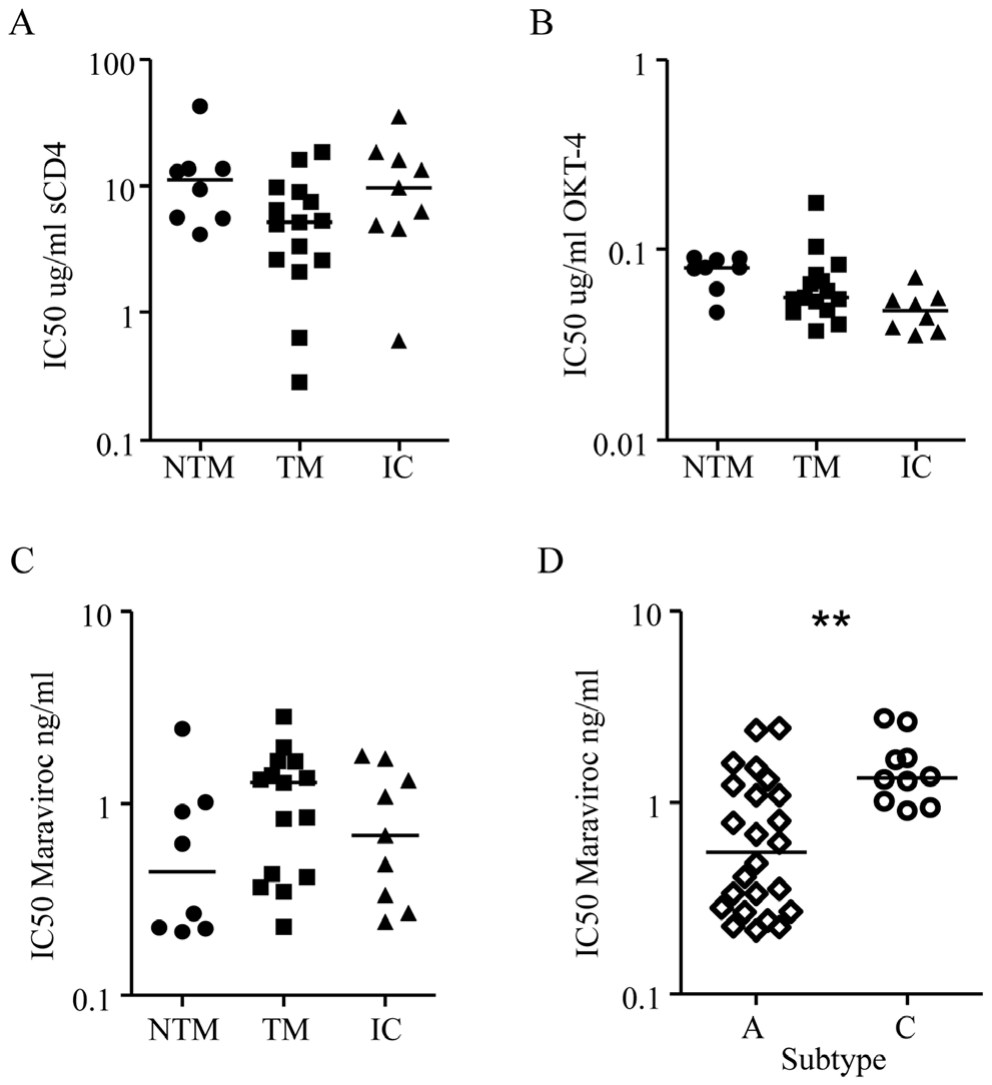
Inhibition of Env pseudo-typed viruses from NTM, TM and infected children (IC) expressed as IC_50_’s. (A) sCD4, (B) αCD4 Ab OKT4 and (C) CCR5 antagonist Maraviroc. NTM are represented by dots, TM by squares, and IC by triangles. Competition with Maraviroc of subtype A (open diamonds) versus subtype C (open dots) viruses is shown in D. **Probability that the groups are similar is <0.01.

We examined the affinity for the CD4 receptor by incubating the selected pseudo-typed viruses with increasing concentrations of sCD4 on TZM-bl cells (Fig. 3A). The median 50% inhibitory concentration (IC_50_) of sCD4 in the pseudo-typed viruses from TM tended to be lower than the median in the NTM (*P = *0.06) suggesting higher affinity for the CD4 receptor in the TM group. The median IC_50_ of sCD4 in TM and children was not significantly different. We also measured the effect of CD4 directed monoclonal Ab OKT4 by incubating TZM-bl cells with increasing concentrations of OKT4 before infection with pseudo-typed viruses (Fig. 3B). No difference was found in the median IC_50_ of OKT4 in the NTM compared with the IC_50_ in TM, but the median IC_50_ in the children tended to be lower than in TM (*P = *0.06), suggesting lower affinity for the CD4 receptor of viruses from children compared to mothers. In conclusion, no evidence was found for transmission selection of viruses with higher affinity for the CD4 receptor in our cohort.

### No Differences in NTM, TM or Children’s virus Interacting with the CCR5 Coreceptor

We next determined whether there were differences in virus coreceptor utilization between the different groups. Initially we determined the coreceptor usage of the Env pseudo-typed viruses by incubating TZM-bl cells with the CXCR4 inhibitor ADM3100, with the CCR5 inhibitor Maraviroc and with both before infection with the selected pseudo-typed viruses. One variant from TM 100 was found to be dual-tropic for CCR5 and CXCR4 whilst all other Env’s from NTM, TM and children were exclusively CCR5 using. We then examined the affinity of the Env pseudo-typed viruses for the CCR5 receptor by incubating TZM-bl cells with increasing concentrations of Maraviroc and determining IC_50_ values (Fig. 3C). The dual-tropic clone of mother 100 was excluded from this analysis. We found no significant differences between the three groups, but the median IC_50_ of subtype A gp160s were significantly lower than for subtype C (*P = *0.006) (Fig 3D). When we analyzed the gp160s of the two subtypes separately no significant differences in IC_50_ values were found between NTM, TM and children. We can conclude that enhanced affinity for the CCR5 coreceptor was not providing selection in MTCT.

### No Differences in Env Interaction with DC-SIGN or Efficiency of virus Transfer from DC-SIGN Bound viruses to TZM-bl Cells from TM, NTM or Children

**Figure 4:**
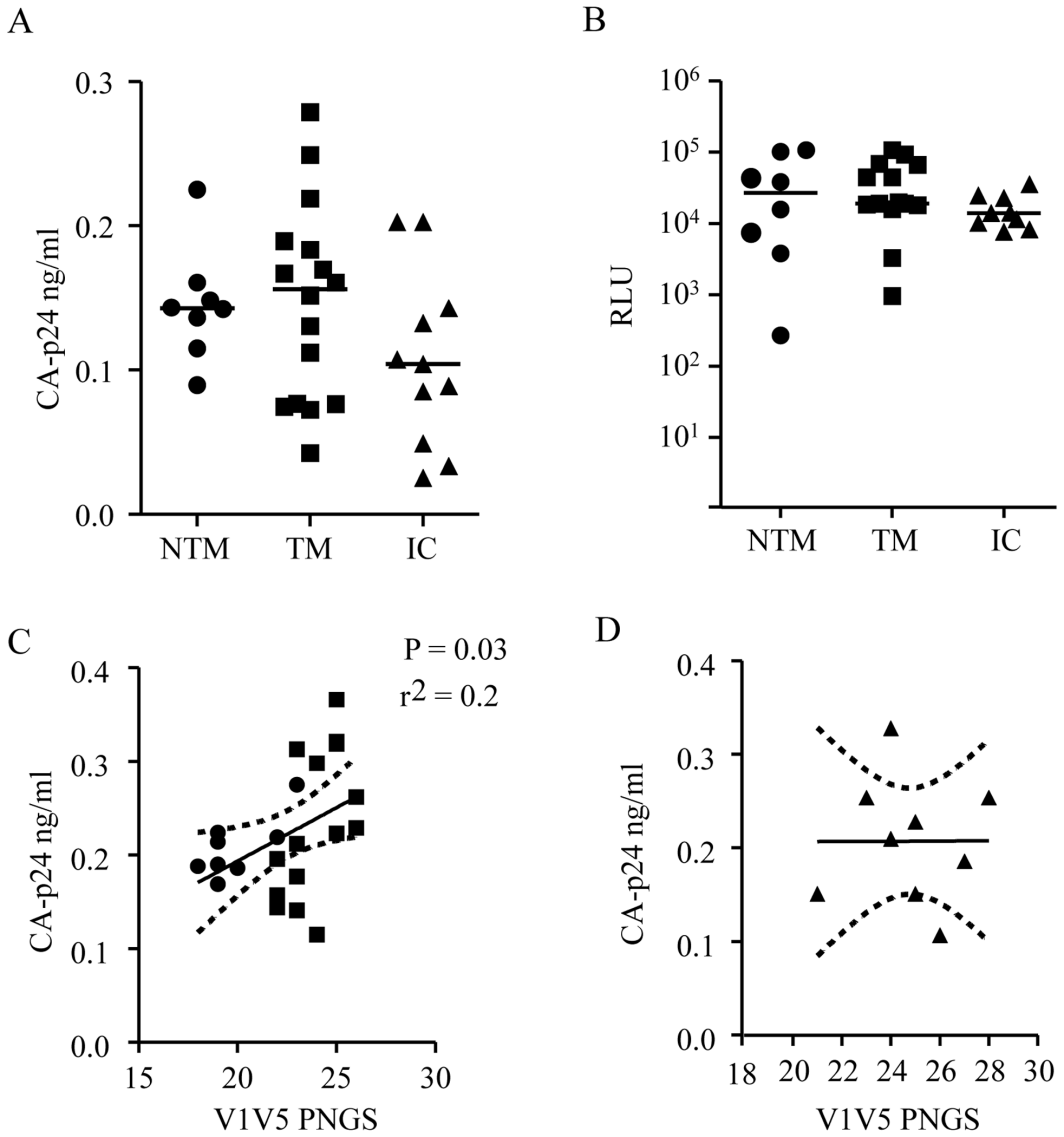
DC-SIGN mediated capture and transfer of Env pseudo-typed viruses from NTM, TM and infected children (IC). (A) Quantity of Env pseudo-typed virus captured by Raji-DC-SIGN cells as determined by CA-p24. (B) Extent of infection of TZM-bl cells by captured Env pseudo-typed viruses expressed in relative luciferase units (RLU). (C and D) Linear regression analysis of the V1–V5 number of PNGS and virus capture by Raji-DC-SIGN cells, in (C) NTM and TM and (D) in IC. NTM are represented by circles, TM by squares, and IC by triangles.

Since it has previously been shown that DC-SIGN polymorphisms can associate with risk of transmission [44] and that factors found in bodily secretions, such as breast milk, can bind this molecule [49] we choose to study how our HIV-1 pseudo-typed viruses interacted with this specific C-type lectin. To determine whether there is a role of DC-SIGN selection in MTCT in our cohort we examined the interaction of the selected pseudo-typed viruses in a DC-SIGN capture assay (Fig. 4A). We found no significant differences between Env pseudo-typed viruses between NTM, TM and children. We subsequently tested the transfer of DC-SIGN captured pseudo-typed viruses to TZM-bl cells to mimic *in vivo* transfer to CD4^+^ lymphocytes (Fig. 4B). We found no differences in infection of transferred pseudo-typed viruses between NTM and TM, but the transfer by children viruses tended to be lower (*P = *0.07) reflecting the lower direct infectivity previously observed with the same viruses (Fig. 2). Since DC-SIGN binding is influenced by the gp120 glycan composition we investigated the correlation between the number of PNGS in the V1V5 region of the pseudo-typed viruses and the levels of CA-p24 captured by DC-SIGN (Fig. 4C and 4D). We found a significant correlation between viruses from TM and NTM (r^2^ = 0.2, *P = *0.03) (Fig. 4C) but no correlation in the clade C viruses suggesting another selection for these glycans opposed to DC-SIGN binding (Fig. 4D). In conclusion we found no evidence of enhanced capture or efficiency of transfer to TZM-bl cells for the viruses undergoing MTCT in our cohort.

### No Difference in Sensitivity of Env Pseudo-typed viruses from TM, NTM or Children to Potent NAbs

Using the TZM-bl neutralization assay we tested the sensitivity profile of our selected pseudo-typed viruses to a panel of well characterized human NAbs; IgG1b12 (b12) directed to the CD4 binding site, glycan dependent 2G12 and the gp41 Membrane Proximal External Repeat (MPER) NAbs 2F5 and 4E10 (Table 2). We found that all pseudo-typed *env* clones were sensitive to neutralization by 4E10. The IgG1b12 epitope was present in the viruses of five transmission pairs (90/91, 130/131, 250/251, 300/301 and 370/371), the viruses of two of them (300/301, 370/371) were resistant to neutralization by b12. Two of the 3 clones of mother 100 and the clone of child 301 were resistant to 2G12 in spite of possession of al the 5 Putative N-Glycosylaton Sites (PNGS) associated with neutralization. The clones of TM 90 and 300 were resistant to 2G12 whilst one of the two clones of child 91 and the clone of child 301 showed sensitivity to neutralization. The subtype C variants of transmission pair 130/131 and NTM 270, with an A→Q mutation at the C terminal of the 2F5 epitope, provided resistance to this NAb. A negative correlation was found between the length of variable loop V4 and the IC_50_ values with 4E10 (r^2^ = 0.152, *P = *0.032) and the IC_50_ values of 2F5 (r^2^ = 0.13, *P = *0.0009) (data not shown). Overall we found no significant differences in neutralization sensitivity between the gp160s of NTM, TM and children with the NAbs tested.

**Table 2:** Neutralization of Env pseudo-typed viruses by panel of Nabs (2G12, b12, 2F5 and 4E10).

Subject	Subtype	Category	Route	2G12 (IC_50_ µg/ml)	B12 (IC_50_ µg/ml)	2F5 (IC_50_ µg/ml)	4E10 (IC_50_ µg/ml)
90P02	A	TM	IU	>30	**3.88**	**14.41**	**4.33**
90P05	A	TM	IU	>30	**4.05**	**13.21**	**5.30**
91P26	A	IC	IU	>30	**2.17**	**11.57**	**2.82**
919342	A	IC	IU	**0.52**	**15.29**	**5.43**	**2.48**
100P37	A	TM	IU	>30	>30	**5.54**	**0.61**
100P21	A	TM	IU	>30	>30	**8.16**	**1.42**
100P32	A	TM	IU	>30	>30	**0.47**	**0.09**
101P19	A	IC	IU	>30	**3.87**	**1.71**	**1.00**
130P17	C	TM	PP/BF	>30	**2.64**	>30	**1.62**
130P16	C	TM	PP/BF	>30	**4.83**	>30	**1.56**
130P10	C	IC	PP/BF	>30	**0.37**	>30	**4.94**
250P10	A/C	TM	IU	**3.26**	**1.93**	**2.52**	**1.41**
250P11	A/C	TM	IU	**0.89**	**4.60**	**1.41**	**2.05**
251P22	A/C	IC	IU	**0.04**	**2.63**	**2.78**	**1.06**
290P04	A	TM	IU	**0.66**	**0.93**	**0.39**	**0.67**
290P01	A	TM	IU	**2.30**	**0.50**	**0.21**	**0.56**
291P187	A	IC	IU	**0.08**	**1.16**	**0.60**	**0.32**
300P29	A	TM	PP/BF	>30	>30	**4.03**	**3.68**
300P31	A	TM	PP/BF	>30	>30	**3.50**	**0.42**
301P91	A	IC	PP/BF	**2.14**	>30	**0.20**	**0.23**
370P23	A	TM	PP/BF	>30	>30	**3.03**	**1.65**
370P19	A	TM	PP/BF	>30	>30	**0.61**	**0.28**
371P131	A	IC	PP/BF	>30	>30	**0.58**	**0.27**
160P40	A	NTM	N/A	>30	>30	**2.92**	**2.88**
160P38	A	NTM	N/A	>30	>30	**1.52**	**1.44**
200P20	A	NTM	N/A	>30	**0.11**	**0.71**	**0.51**
200P22	A	NTM	N/A	>30	**0.02**	**1.84**	**0.98**
270P03	C	NTM	N/A	>30	**1.86**	>30	**3.79**
270P07	C	NTM	N/A	>30	**1.25**	>30	**2.03**
380P02	A	NTM	N/A	**0.56**	**0.03**	**0.42**	**0.24**
380P04	A	NTM	N/A	**1.36**	**0.04**	**0.62**	**0.87**

TM; transmitting mother, NTM: non-transmitting mother, IC: infected child, IU: *in utero*, PP: *peri-partum*, BF: breastfeeding, **Bold:** IC_50_<30.

### Strong Autologous Neutralization by Plasma from TM and Infected Children

**Figure 5:**
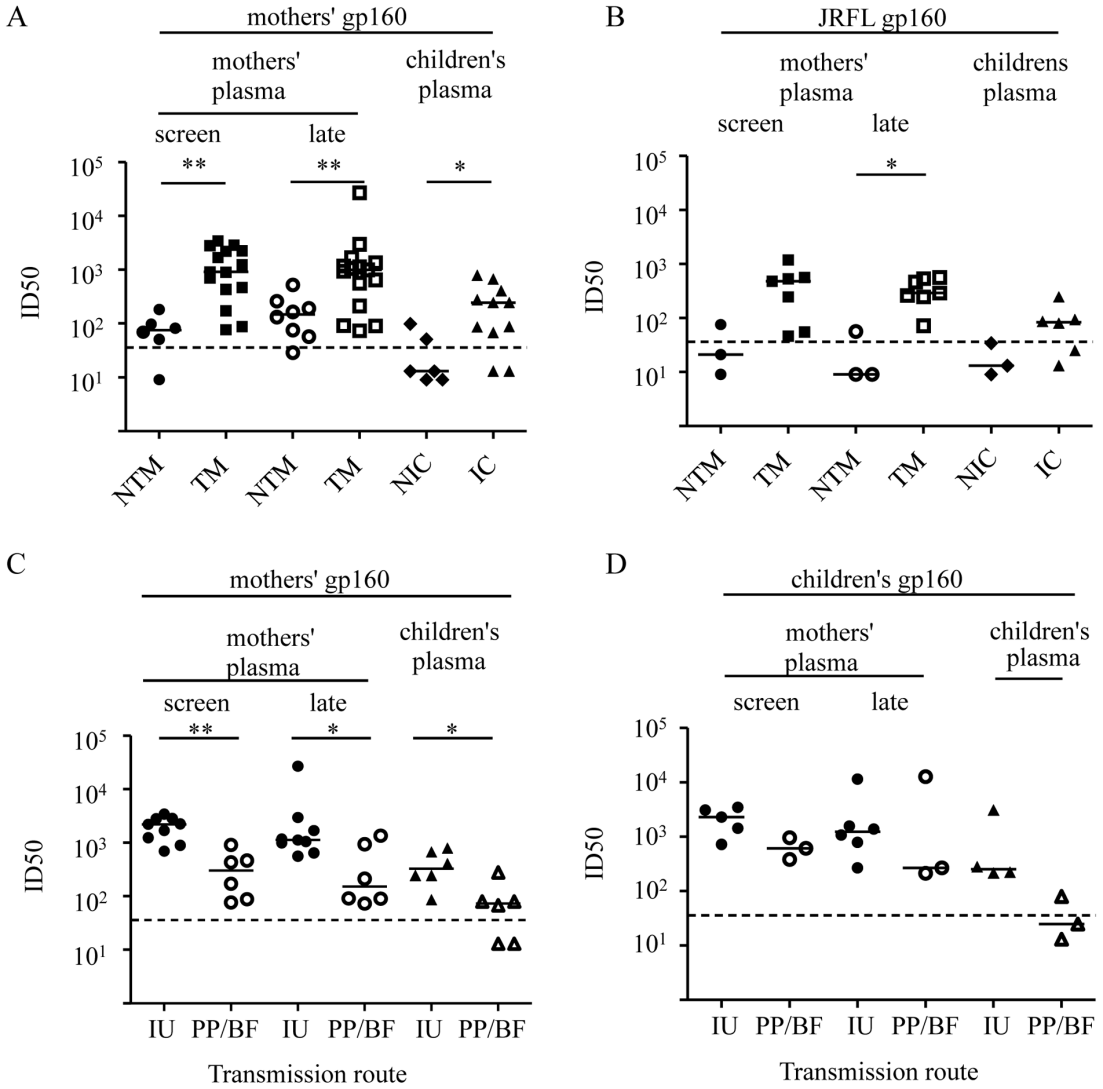
Plasma neutralization of NTM, TM, infected children (IC) and non-IC. (A) Neutralization of Env pseudo-typed viruses carrying NTM and TM gp160. (B) Neutralization of Env pseudo-typed viruses carrying the JRFL gp160. Neutralization from NTM is represented by dots, from TM by squares, from NIC by diamonds and from IC by triangles. (C) Autologous neutralization of Env pseudo-typed viruses carrying TM gp160/gp41, divided into IU and PP/BF and (D) neutralization of children’s Env pseudo-typed viruses. Neutralization by plasma from IU TM is represented by closed dots, from PP/BF TM by open dots, from IU IC by closed triangles and from BF IC by open triangles. The dashed line indicates the lowest plasma dilution tested (1→48). *Probability that the groups are similar is <0.05, **Probability that the groups are similar is <0.01.

We next tested the autologous neutralization by plasma from mothers collected one week *pre-partum* (before nevirapine was given and termed “screen”) and between 16 and 18 weeks *post-partum* (termed “late”) as well as corresponding plasma from both infected and uninfected children. For these assays we utilized the corresponding mothers pseudo-typed (Fig. 5A) and JRFL pseudo-typed (Fig. 5B) virus strains. Plasma of week 16 from one IU infected child (101) and one uninfected child (161) were missing. There were no differences observed between TM and NTM in the concentration of overall IgG in plasma’s (data not shown). There was also no correlation observed between viral load and the autologous neutralization by plasma’s (*P = *0.78, r^2^ = 0.004) (data not shown). We found that plasma from TM was significantly better in neutralizing autologous gp160 than the NTM (*P = *0.002 for screen plasma and *P = *0.006 for late plasma) and that plasma from infected children neutralized better than plasma from uninfected children (*P = *0.02). The JRFL gp160 was also better neutralized by TM than by NTM (*P = *0.07 for screen plasma and *P = *0.02 for late plasma) but the 50% inhibitory dose (ID_50_) was 0.5 to 0.9log lower than in the autologous neutralization experiments. In conclusion, autologous plasma from both TM and infected children neutralized gp160s from the corresponding mothers more efficiently than plasma from the NTM and the uninfected infants.

### Neutralization of Plasma more Evident in IU Transmissions than in PP/BF Transmissions

To further investigate differences in neutralization activity between the different transmission routes, we analyzed separately the viruses of the mothers who transmitted IU and those who transmitted PP or BF. We tested neutralization using autologous screen plasma and late plasma as well as plasma from the corresponding child (Fig. 5C). We found significantly better neutralization of the gp160s of the IU TM than of the PP/BF TM by screen plasma (*P = *0.002), by late plasma (*P = *0.03) and by children’s plasma (*P = *0.02). We also analyzed the neutralization of the IU and PP/BF infected children’s *env* clones by screen plasma and late plasma of the corresponding mothers (Fig. 5C) and by autologous plasma (Fig. 5D). Screen plasma from the PP/BF transmitting mothers tended to neutralize the gp160s of children less than plasma from the IU TM (*P = *0.07), but no difference in neutralization was found with the late plasma’s. Autologous gp160s of the corresponding child were better neutralized by screen plasma from PP/BF TM than from NTM (*P = *0.06 and *P = *0.02) (data not shown). The IU infected children’s plasma tended to neutralize autologous gp160s better than the PP/BF infected children (*P = *0.06). At the lowest plasma dilution tested (1∶36) 2/3 PP/BF transmitted viruses could not be inhibited to 50% while 1/3 was 50% inhibited at a dilution of 1∶80, indicating possible immune escape of these viruses. In conclusion we found that viruses of IU transmission pairs were better neutralized than those of PP/BF transmissions.

### No Difference in IgG Binding to Subtype B gp140 Envelope Trimer between Plasma’s from TM and NTM

To rule out that differences in total IgG confused our results, we measured the concentration of IgG in the plasma’s of mothers and children. We found no difference between TM and NTM but the HIV-1 infected children demonstrated higher levels of IgG in their plasma than the uninfected children (12,6 g/l and 5.7 g/l, *P = *0.02) (data not shown). We also investigated the levels of IgG that binds to gp140 envelope trimer in an ELISA assay. To enhance the statistical power of the experiment we included 14 additional plasma samples from NTM from our cohort. We found no differences between TM and NTM. Similar results were obtained with a subtype A gp140 trimer. We also investigated the gp140 binding properties of plasma of the infected children extended with 21 uninfected children from our cohort and again no differences were found (data not shown).

### Multi-variate Analysis of *env* Sequences

In an earlier report we analyzed the gp120 *env* sequences for TM, NTM and infected children and identified two PNGS (N234 and N339) which associated with viruses that preferentially underwent MTCT [20]. We performed a multi variable analysis to test correlations of the presence of one or both PNGS with infectivity, CD4 interaction, CCR5 interaction, DC-SIGN interaction and neutralization sensitivity. We could not identify significant associations with alterations within these sites and the phenotypes tested (data not shown).

## Discussion

We studied the viral and immunologic correlates of the HIV-1 subtype A or C or A/C recombinant gp160 envelope proteins from seven mother/child pairs whose children were infected either during gestation or soon after birth and from four NTM with viral loads and CD4 counts in the same range. We found no viral correlates that could have facilitated HIV-1 MTCT, but TM demonstrated higher plasma neutralization capacity than NTM. IU TM neutralized their autologous Env pseudo-typed viruses and those of their infants to significantly higher levels than mothers that transmitted HIV-1 either PP or via BF. Furthermore, plasma from infants infected IU were able to neutralize their autologous Env pseudo-typed viruses as well as those from their mothers to higher levels in comparison to those infected PP/BF, who showed no autologous neutralization capacity.

It has been hypothesized that HIV-1 variants with a replication fitness advantage are those more likely to be transmitted or dominate and selectively outgrow upon infection. Although replication capacity cannot be directly extrapolated from infectivity, a study using single-cycle viruses from subtype C infected MTCT pairs has reported that such viruses from children have a higher fitness than those generated from the mothers and that this is restricted to the V1V5 region of the envelope [15]. Earlier studies found no difference in infectivity between mothers and children’s gp160s tested in a single-cycle assay on TZM-bl cells [21], [23]. We found here that viruses derived from children were less infectious for TZM-bl cells than those derived from their mothers. This may reflect differences in time of sampling. We studied viruses soon after transmission whereas other studies were conducted with viruses amplified later allowing time for viruses to adapt and gain fitness. Additionally, the discrepancies observed may be partially explained by differences in the cell lines used for pseudo-virus production and the envelope/backbone ratio used in generating the pseudo-typed viruses [50], [51].

The vast majority of HIV-1 infections are initiated by a single or a limited number of viral variants, irrespective of transmission route or HIV-1 subtype, indicating a severe bottleneck to transmission [17], [52]. X4 viruses are rarely transmitted. The variants undergoing both horizontal as well as MTCT are mainly R5 [4], [5], [53]. Whether this results from selection of R5 viruses, the early replication outgrowth of R5 over X4 strains or the limited presence of X4 viruses at the site of exposure is still unknown [54]–[56]. A higher affinity for HIV-1 binding to CD4 or the CCR5 coreceptor may provide a level of selection, however, in concordance with earlier publications studying horizontal as well as vertical transmission cases we did not find differences between transmitted and not transmitted variants in ability to interact with either CD4 or CCR5 [13], [14], [16].

HIV-1 MTCT requires virus to cross a mucosal barrier, be it via the placenta or the oral/gastrointestinal tract. Mucosal infection can be heightened through the interaction of HIV-1 with an array of C-type lectins, including DC-SIGN, expressed on DCs lying below the mucosal epithelium [41]–[43]. DC-SIGN allows for the capture of virus and its heightened transfer to CD4^+^ lymphocytes via *trans-infection*. We found no difference in the efficiency of DC-SIGN capture and transfer of virus to TZM-bl cells between NTM, TM and children. A positive correlation, however, was found between the number of PNGS with DC-SIGN capture when analyzing *env* clones from TM as well as NTM but not for viruses from children. This suggests that viruses transferred to children are not selected based on their capacity for binding DC-SIGN. It should be noted that the pseudo-typed viruses studied were produced within the human C33A cell line and therefore likely possess the relevant post-translational glycosylation modifications relevant to the human *in vivo* situation [50], [57]. Furthermore, other C-type lectins expressed on DCs can capture HIV-1, however here we specifically focused on DC-SIGN since genetic polymorphisms within this molecule have been associated with risk of transmission [44]. It remains to be assessed whether other molecules expressed on DCs can associate with heightened viral capture and transfer and thereby contribute to MTCT.

HIV-1 subtype B R5 Env variants derived from acutely infected individuals via horizontal transmission have been shown to possess NAb inhibitory profiles similar to those derived from chronically infected individuals [58]–[61]. However, other studies analyzing viruses undergoing sexual transmission indicate that these variants encode gp120s with a compact structure and a reduced number of PNGS that are more sensitive to the effects of NAbs [17], [59]. Sensitivity to NAbs may therefore not be a disadvantage in horizontal transmission where the recipient is Ab naïve. In MTCT the situation is different where the child inherits HIV-1 Abs from the mother. It has recently been shown that NAbs can provide selection for a gp120/gp41 envelope with the capacity to induce more potent and broadly neutralizing Abs [62]. Here we found no differences between the viruses from mothers and children for inhibition with the broadly NAbs b12, 2G12, 2F5 and 4E10, suggesting no such selection regarding envelope structure.

Reports studying mother child pairs have shown better neutralization by NTM than by TM suggesting a protective role by Abs [24], [25]. Here we found no such effect and actually identified a stronger neutralization profile of autologous viruses within the TM opposed to NTM group. In accordance, we found no neutralization of JRFL with plasma from NTM whilst we did observe neutralization of the same virus with plasma from NTM. One limitation to our study, and inherent to all such studies, is that the exact timings of transmission are not known and where viral adaptations can occur. When we compare the TM to NTM neutralizations of either autologous virus or JRFL the same effect is seen for both early and late plasma from mothers and where the neutralization of viruses from children, all amplified from a time point between plasma sampling of the mother, show similar results. These findings collectively indicate that the observed effect of heightened neutralization is unlikely to be due to differences in timings of transmissions and the subsequent development of escape or reduced neutralization sensitivities. Another limitation to our study is the low number of mother child pairs where HIV-1 transmission occurred and especially when considering sub-groupings. However, it is clear that autologous NAb responses, in either the mother or infant, do not protect against MTCT, and may in fact be detrimental when considering IU transmissions. We highlight that additional studies of larger cohorts need to be undertaken to confirm such conclusions, but our results indicate that autologous NAb responses are not protective.

In both PP and BF transmission HIV-1 has to cross the oral or intestinal mucosal layer. *Ex vivo* experiments have shown that HIV-1 infected macrophages and to a lesser extent HIV-1 lymphocytes are able to migrate across the oral and intestinal epithelium [40]. For IU transmission the virus has to transgress the trophobastic layer that separates the placenta from the fetal blood and the amniotic fluid. Experiments were performed with the BeWo polarized trophoblast cell line that mimics the barrier between maternal and fetal blood and between placenta and amniotic fluid [35]. As shown by these experiments PBMCs, lymphocytes and macrophages are able to fuse to the apical side of the trophoblasts, resulting in release of infectious virus at the basal lateral side of the monolayer in parallel with infection through the mucosa of the fetal digestive tract [35].

During gestation a considerable amount of maternal IgG is transferred to the fetus and additionally IgG is transferred to the newborn child by breastfeeding. This process is mediated by the FcRn receptor expressed on trophoblasts and intestinal mucosa which binds maternal IgG followed by transcytosis and release at the basal side of the mucosa into the fetal blood, into the amnion fluid or the digestive tract of the child [35], [63]. This mechanism may provide a means whereby HIV-1 in IgG/HIV-1 complexes can bind FcRn and be transported to the fetus or the newborn child. This process will be favored by higher concentrations of Env binding IgG. Although others have reported stronger binding (gp160, V3 and gp41 directed) IgG Abs in TM opposed to NTM we found higher levels of NAbs favor MTCT [37], [38]. The exact conformation of the HIV-1/IgG complex that is able to bind in the proper manner to FcRn and thereby be transcytosed may have restrictions, steric or otherwise. High affinity between IgG and the virus particle may therefore be significant for both neutralizing activity as well as for FcRn transcytosis of the virus-IgG complex. Ab dependent enhancement of HIV-1 binding and infection of certain cell types has been previously demonstrated *in vitro* and may indeed explain for our observed findings [45], [64]–[66].

We demonstrate here that in the cohort we investigated the neutralizing function of maternal Abs, rather than preventing, associates with a higher risk of IU MTCT. In future studies it would therefore be prudent to consider the different routes of exposure when comparing immunologic factors associated with MTCT, in both the mother and the infant. These results indicate that different mechanisms may support viral transmission via the variant routes of exposure. More so, our results suggest caution when considering using NAbs for passive immunization to prevent IU MTCT and in inducing high levels of NAbs in pregnant vaccine recipients. Larger studies need to be performed to support these findings and especially to characterize the specificities and properties of such induced Abs and to understand which responses can be beneficial opposed to being detrimental.

## Methods

### Study Population and Ethics Statement

The study was approved by an Independent Ethics Committees (IEC) in the Netherlands, the STEG-METC (ref no R01–089). In the absence of an operational IEC in Rwanda at the time, the Ministry of Health’s Treatment and Research AIDS Center (TRAC), the Rwandan National Malaria program (PNLP) and the “Cellule de recherché” at the CHK acknowledged the approval of the Dutch IEC. All women provided written informed consent for both themselves and their children. Plasma samples were utilized from HIV-1 positive mothers (n = 30) and their infants from Rwanda [20]. All women received single dose Nevirapine at onset of labor. Maternal plasma was collected prior to delivery (screen), at labor (early) and week 16 and 18 (late) after delivery. Child’s plasma was collected at delivery (early) and 16 weeks (late) of age. All children were breastfed during the 16 week study period.

### HIV-1 RNA Extraction and Amplification

HIV-1 RNA was isolated from plasma samples according to the method of Boom *et al* [67]. Viral RNA was converted to cDNA as previously described [6] with an input of 6,000 copies RNA per amplification. We selected this copy number to limit recombination events that can arise during PCR amplification and to minimize the risk associated with such an approach. Reverse transcription of RNA to single-stranded cDNA was performed using the SuperScript III protocol according to the manufacturer’s instructions (Invitrogen Life Technologies, Carlsbad, CA) with a modification. RNA, Betaine (1 M) deoxynucleoside triphosphates (0.5 mM each), and 0.25 µM primer OFM19 (5′-GCACTCAAGGCAAGCTTTATTGAGGCTTA-3′; nucleotides [nt] 9604 to 9632 of the HXB2 sequence) were incubated for 5 min at 65°C to denature secondary structure of the RNA. First-strand cDNA synthesis was carried out in 20 µl reaction mixtures with First-Strand Buffer containing 5 mM dithiothreitol, 2 U/µl of an RNase inhibitor (RNaseOUT), and 10 U/µl Super- Script III. The reaction mixture was incubated at 55°C for 65 min and inactivated by being heated to 65°C for 15 min. The resulting cDNA was used immediately for PCR.

### Gene Amplification

Full-length *rev/env* genes (including parts of the first exon of the *tat* gene; the entire *vpu*, *rev*, and *env* genes; and parts of the *nef* gene) were amplified by nested PCR from plasma-derived viral cDNA as previously described with minor changes [6]. Briefly, 20 µl of bulk cDNA (containing 100 to 1,000 viral templates) was subjected to first-round PCR in a volume of 50 µl. PCR was performed by using an Expand High Fidelity PCR system (Roche Diagnostic Corporation, Indianapolis, IN) in 5µl Expand PCR buffer containing 1.5 mM MgCl_2_, 0.2 mM of each deoxynucleoside triphosphate, and 0.2 µM of Vif1 (5′-GGGTTTATTACAGGGACAGCAGAG-3′; nt 4900 to 4923) and OFM19 primers. The following cycling conditions were used: 93°C for 2 min followed by 10 cycles of 93°C for 10 s, 58°C for 30 s, and 68°C for 5 min, and followed by 20 cycles of 93°C for 10 s, 58°C for 30 s, and 68°C for 5 min extended with 20 s with each cycle, with a final extension of 68°C for 7 min. Second-round PCR was performed by using 5 µl of the first-round PCR product and primers EnvA (5′ GGCTTAGGCATCTCCTATGGCAGGAAGAA-3′; nt 5954 to 5982) and EnvN (5′-CTGCCAATCAGGGAAGTAGCCTTGTGT-3′; nt 9145 to 9171) under the same conditions used for the first-round PCR.

### Construction of Envelope Clones

The final PCR products of the predicted size (±3.2 kb) were ligated into the pcDNA3.1.V5-His TOPO TA vector according to the manufacturer’s instructions (Invitrogen Life Technologies, Carlsbad, CA) and transformed into Stbl4 cells by electroporation (Invitrogen Life Technologies, Carlsbad, CA). Reaction mixtures were plated on LB-Amp (ampicillin; 100µg/ml) plates and cultured 24 h at 30°C. Colonies were screened with a colony PCR using primers T7 (5′-TAA TAC GAC TCA CTA TAG GG A-3′) and FGS021 (5′-CTT TCA TTG CCA CTG TCT TCT GCT-3′). Clones containing the right size and orientation were grown in LB-Amp (ampicillin; 100 µg/ml) and plasmid was isolated with the Bioké NucleoSpin Kit (Machery-Nagel, Düren, Germany). The plasmids were screened by generating Env pseudo-typed virus and tested for infectivity as described below and the infectious clones were selected.

### Cells and Transfections

C33A cells and TZM-bl cells (obtained through the NIH ARRRP from Dr. John C. Kappes, Dr. Xiaoyun Wu and Tranzyme Inc., Durham, NC) were maintained in Dulbecco’s modified Eagle’s medium (DMEM; Invitrogen, Breda, The Netherlands), each supplemented with MEM nonessential amino acids and 10% foetal calf serum (FCS), penicillin (100 U/ml) and streptomycin (100 µg/ml) as previously described (58). One day prior to transfection 2.5×10^6^ C33A cells were seeded in a 25 cm^2^ culture flask. After 24 h the cells were transfected using the CaPi method. In short: HEPES-buffer (50 mM HEPES pH7.1, 250 mM NaCl, 1.5 mM Na_2_HPO_4_) was added to 9 µg of the envelope containing plasmids and 3.9 µg of plasmid pSG3Δ*env* in a volume of 293 µl, mixed well and 40 µl of 2M CaCl_2_ was added and again mixed well. After 45 min incubation at rt the mixture was added to the C33A cells. As a positive control JRFL envelope was used and pSG3Δ*env* alone for the negative control. After 18 h the medium was refreshed and the pseudo-typed virus containing supernatant was harvested after 48 h of incubation. Virus was stored at −80°C. The virus concentration was quantified by measuring its CA-p24 antigen content by ELISA.

### Sequencing and Phylogenetic Analysis

The infectivity competent *env* clones were sequenced using the ABI PRISM Big Dye Terminator Kit (Applied Biosystems, Forster City, CA, USA). Sequences were assembled using the CodonCode Aligner program (CodonCode Corporation, Dedham, USA). Alignments were performed taking the translation codons into account and the sequences were translated into amino acids using the BioEdit program (Tom Hall, Ibis Therapeutics, Carlsbad, CA, USA). Phylogenetic analysis of the envelopes was performed with the Neighbor-Joining (N-J) method of MEGA version 4 (Tamura, Dudley, Nei, and Kumar 2007). Positions containing an alignment gap were included for pair wise sequence analysis**.** The number PNGS was determined using the program available at the HIV Sequence Database (http://www.hiv.lanl.gov/content/sequence/GLYCOSITE/glycosite.html). The gp160 envelope sequences have been submitted to GenBank under accession numbers JX508855 - JX508885.

### Single-cycle Infections and Inhibition Assays

One day prior to infection, 17×10^3^ TZM-bl cells were added to a 96-well plate and cultured overnight. Infections were performed in 200 µl with 1 pg CA-p24 virus input and medium supplemented with 10µg/ml DEAE dextran. Two days post-infection the medium was removed, the cells were washed once with PBS and lyzed in reporter lysis buffer (Promega, Madison, WI). Luciferase activity was measured using the Luciferase Assay kit (Promega) and a Glomax luminometer according to the manufacturer’s instructions (Turner BioSystems, Sunnyvale, CA). All infections were performed in duplicate and repeated at least once. Cells infected with negative control pseudo-typed virus were used to correct for background luciferase activity. The infectivity of each mutant without inhibitor was defined as 100%. Non-linear regression curves were determined and IC_50_ or ID_50_ values were calculated using Inhibitor versus Response variant of the Non Linear Regression function of GraphPad Prism version 5.00 for Windows, (GraphPad Software, San Diego California USA, www.graphpad.com) and a significance level of *P*<0.05 was used for all the analyses.

The program Factor Correction was used to remove multiplicative between-session variation [68]. For determination of the infectivity of the pseudo-typed viruses three step serial dilutions of virus were used. In inhibition experiments an amount of virus equal to 1pg of p24/well was used. In experiments with Maraviroc or OKT-4 (eBioscience, San Diego, CA) TZM-bl cells were incubated with 3 step dilutions of inhibitor for 30 min at 37° before the pseudo-typed virus was added. For inhibition with sCD4-183 pseudo-typed virus was incubated with 3 step dilutions for 30 min at 37° before infection of the cells. The maximum concentration of sCD4, Maraviroc and OKT4 was 10 µg/ml, 10 ng/ml and 30 ng/ml respectively.

### Virus Capture and Transfer by Raji-DC SIGN cells

For the DC-SIGN capture and transfer experiments 50 µl of Raji or Raji-DC-SIGN cells (20×10^6^ cells/ml) were mixed with 150 µl of pseudo-typed virus equal to 50 ng/ml CA-p24 and incubated for 2 h at 37°, washed 4× with ice cold PBS and resuspended in 90 µl of culture medium. 10 µl of this suspension was used in the CA-p24 ELISA to quantify the amount of virus captured by DC-SIGN, corrected for non-specific binding to the Raji cells. 15 µl of the suspension was used to infect TZM-bl cells. After 48 h the TZM-bl cells were lyzed and luciferase activity was measured as described above.

### HIV-1 Neutralization

Assays measuring HIV-1 inhibition using NAbs b12, 2F5, 4E10 and 2G12, maternal or children’s plasma were performed as previously described [69], using virus input equal to 1 pg CA-p24 of pseudo-typed virus/well. Before infection the virus was incubated with 3 step dilutions of NAb or plasma for 30 min at 37°. The maximum Ab concentration used in neutralization assays was 10 µg/ml. The lowest dilution of plasma was 1∶36. All plasma samples were heat inactivated at 56°C for 30 min before use. Plasma from a seronegative donor was used as a negative control and showed no neutralization activity at 1∶36 dilution.

### Determination of IgG Concentrations

IgG concentrations in plasma were measured using the Cobas C502 (Roche, Roche Diagnostics, Darmstadt, Germany) according to the manufacturers instructions.

### gp140 Trimer Binding ELISA

The ELISA was performed as earlier described [70], [71]. Microlon 96 well plates (Greiner Bio-One, The Netherlands) were coated overnight with D7324 Ab 100× diluted in 0.1 M NaHCO_3,_ pH 8.6 and washed 2× with TBS (150 mM NaCl, 10 mM TRIS, pH 7.5). SOSIP.R6-IZ-D7324 trimer diluted in TBS/10% FCS was added and incubated for 2 h at RT, and washed 2× with TBS followed by 1 hr incubation with 2% skimmed milk in TBS. Serial dilutions of plasma in TBS/2% milk/20% sheep serum were added and incubated for 2 h followed by 5 washes with TBS/0.05% Tween-20. Goat- anti-human Fc-HRP (Jackson Immuno Research, England) 1∶1000 diluted in TBS/2% milk/20% sheep serum was added and incubated for 30 min. followed by 5 washes with TBS/Tween. All reactions were performed in 100 µl/well. Colorimetric detection was performed by adding 50 µl of 1%TMB (Sigma-Aldrich, the Netherlands), 0.01% H_2_O_2_, 1 M Sodium Acetate, 0.1 M citric acid and the reaction was stopped by adding 50 µl 0.8 M H_2_SO_4_/well when appropriate and absorption at 450 nm was measured.

### Statistical Analysis

To avoid confounding of the analysis caused by the use of more than one data point derived from each of the members in the groups of NTM, TM and infected children we performed the Kruskall-Wallis test to determine within and between individual differences in our results on infectivity, CD4 interaction, CCR5 interaction, DC-SIGN interaction and neutralization sensitivity and with a Bonferroni correction for multiple testing. No significant differences were found between the individuals within each group and therefore the Mann-Whitney test was used to analyze the differences between the groups. Analysis of the association of the presence of one or both PNGS N234 and N339 with infectivity, CD4 interaction, CCR5 interaction, DC-SIGN interaction and neutralization sensitivity was done with the multivariate logistic regression analysis using PASW 17.0. hypotheses testing, all variables were introduced through forward stepwise procedure. A significance level of *P*<0.05 was used for all the analyses.
